# Prognostic Value of Bcl‐xL in Head and Neck Squamous Cell Carcinoma: A Systematic Review and Meta‐Analysis

**DOI:** 10.1111/jop.70155

**Published:** 2026-06-13

**Authors:** L. Leinonen, R. Tiikkaja, M. Juola, P. Åström, T. Salo, K. Juurikka, K. Korelin

**Affiliations:** ^1^ Research Unit of Population Health, Faculty of Medicine University of Oulu Oulu Finland; ^2^ Medical Research Centre Oulu University Hospital Oulu Finland; ^3^ The Wellbeing Services County of North Ostrobothnia Oulu Finland; ^4^ Research Unit of Biomedicine and Internal Medicine, Faculty of Medicine University of Oulu Oulu Finland; ^5^ Biocenter Oulu Oulu Finland; ^6^ Department of Oral and Maxillofacial Diseases University of Helsinki, Helsinki University Central Hospital Helsinki Finland; ^7^ HUSLAB, Department of Pathology Helsinki University Central Hospital, University of Helsinki Helsinki Finland; ^8^ Translational Immunology Research Program, Faculty of Medicine University of Helsinki Helsinki Finland; ^9^ iCAN Digital Precision Cancer Medicine Flagship University of Helsinki Helsinki Finland

**Keywords:** apoptosis, Bcl‐xL, HNSCC, lymph node metastasis, survival

## Abstract

**Background:**

Head and neck squamous cell carcinoma is a heterogeneous disease with poor survival outcomes. Bcl‐xL regulates tumor cell survival and apoptosis and has been associated with metastasis and poor prognosis, although its overall role remains undeciphered.

**Methods:**

We systematically evaluated the prognostic value of Bcl‐xL in head and neck squamous cell carcinoma following PRISMA 2020 guidelines. We identified 2241 reports from seven databases, of which 20 original studies with overall good quality (REMARK) and low bias (QUIPS) were included.

**Results:**

An association between Bcl‐xL levels and survival was rarely observed. However, high Bcl‐xL levels correlated with increased risk of lymph node metastasis in 40% of studies. A study on oral tongue cancer identified a significant correlation between high Bcl‐xL levels and decreased survival and lymph node metastasis.

**Conclusions:**

Current evidence does not support Bcl‐xL as a general prognostic marker in head and neck squamous cell carcinoma. Nevertheless, Bcl‐xL may have prognostic relevance in oral tongue cancer if further studies confirm the original finding.

## 1 Introduction

Head and neck squamous cell carcinoma (HNSCC) is the sixth most common cancer worldwide, resulting in 890 000 new cases and 460 000 deaths in 2022 [1]. Currently, the 5‐year overall survival (OS) rate for HNSCC is roughly 65%, but varies substantially by cancer subsite and subtype [2]. HNSCCs are derived from the mucosal epithelium of the upper aerodigestive tract, including the oral cavity, nasopharynx, oropharynx, hypopharynx, and larynx. Alcohol consumption, tobacco smoking, and human papilloma virus (HPV) represent common risk factors for the oncogenic transformation of epithelial cells and for HNSCC development [3, 4, 5].

HNSCC subtypes exhibit significant heterogeneity, whereby their tumor growth is driven by diverse, dysregulated signaling pathways. Understanding the biochemical and genetic profiles of these tumors can offer biomarkers to targeted therapies for each HNSCC subtype [6]. A biomarker is a biomolecule, such as DNA, RNA, a protein, a peptide, or a biomolecule chemical modification, which describes a normal or abnormal biological state in an organism that can be objectively measured [7]. Various types of HNSCC have potential tumor tissue–specific biomarkers, representing potential therapeutic targets. Yet, resistance to targeted therapies is common among HNSCC patients and thus crucial to explore in order to develop more effective and tolerable therapies [8].

Abnormalities in the expression of apoptosis pathway factors, such as B‐cell lymphoma‐2 (Bcl‐2) family members, can lead to defects in the control of programmed cell death and promote the development of and therapy resistance in cancer. Members of the Bcl‐2 protein family, which regulate the intrinsic pathway of apoptosis, are classified into three subgroups based on their Bcl‐2 homology (BH) domains: pro‐survival proteins (e.g., Bcl‐2, Bcl‐xL), pro‐apoptotic BH3‐only proteins (e.g., BAD, BID), and effectors of apoptosis (BAX, BAK, and BOK) [9, 10]. The BCL2 Like 1 gene (*BCL2L1*) encodes for a 233‐amino acid–long B‐cell lymphoma‐extra large (Bcl‐xL) protein [11]. Bcl‐xL inhibits the intrinsic apoptosis pathway in multiple ways, including by inhibiting the binding of pro‐apoptotic BAX to the mitochondrial outer membrane. This process inhibits the uptake of calcium to the mitochondria, thereby preventing mitochondrial membrane rupture and the release of cytochrome c from the mitochondria. Moreover, a high Bcl‐xL expression correlates with an increased invasiveness and therapy resistance in many cancers [12].

Studies have shown that the Bcl‐xL protein is consistently upregulated in HNSCC, indicating its potential as an attractive therapeutic target [13, 14]. Accordingly, a high Bcl‐xL expression associates with positive nodal disease and a lower overall survival (OS) in patients with oropharyngeal SCC [15]. Carter et al. [14] demonstrated that HNSCC depends on Bcl‐xL and other anti‐apoptotic proteins for survival, possibly serving as an efficient target to induce marked apoptosis and tumor shrinkage. This suggests that a combination BH3 mimetic therapy may offer significant treatment benefits in HNSCC [14]. Furthermore, we recently reported that, in addition to apoptosis induction, Bcl‐xL inhibitors reduce HNSCC cell proliferation and metastasis in vitro and in zebrafish larvae xenograft assays [16].

While Bcl‐xL appears to play a crucial role in the progression of HNSCC, its prognostic value has not yet been systematically evaluated. In this study, we synthesize the available literature on the association between expression levels of Bcl‐xL and patient survival outcomes as well as the occurrence of lymph node metastases in HNSCC. Bcl‐xL represents a promising biomarker that may help address the critical need for effective prognostic and therapeutic targets for HNSCC.

## 2 Results

### 2.1 Twenty Good Quality Articles Were Identified by Our Search Strategy

**FIGURE 1:**
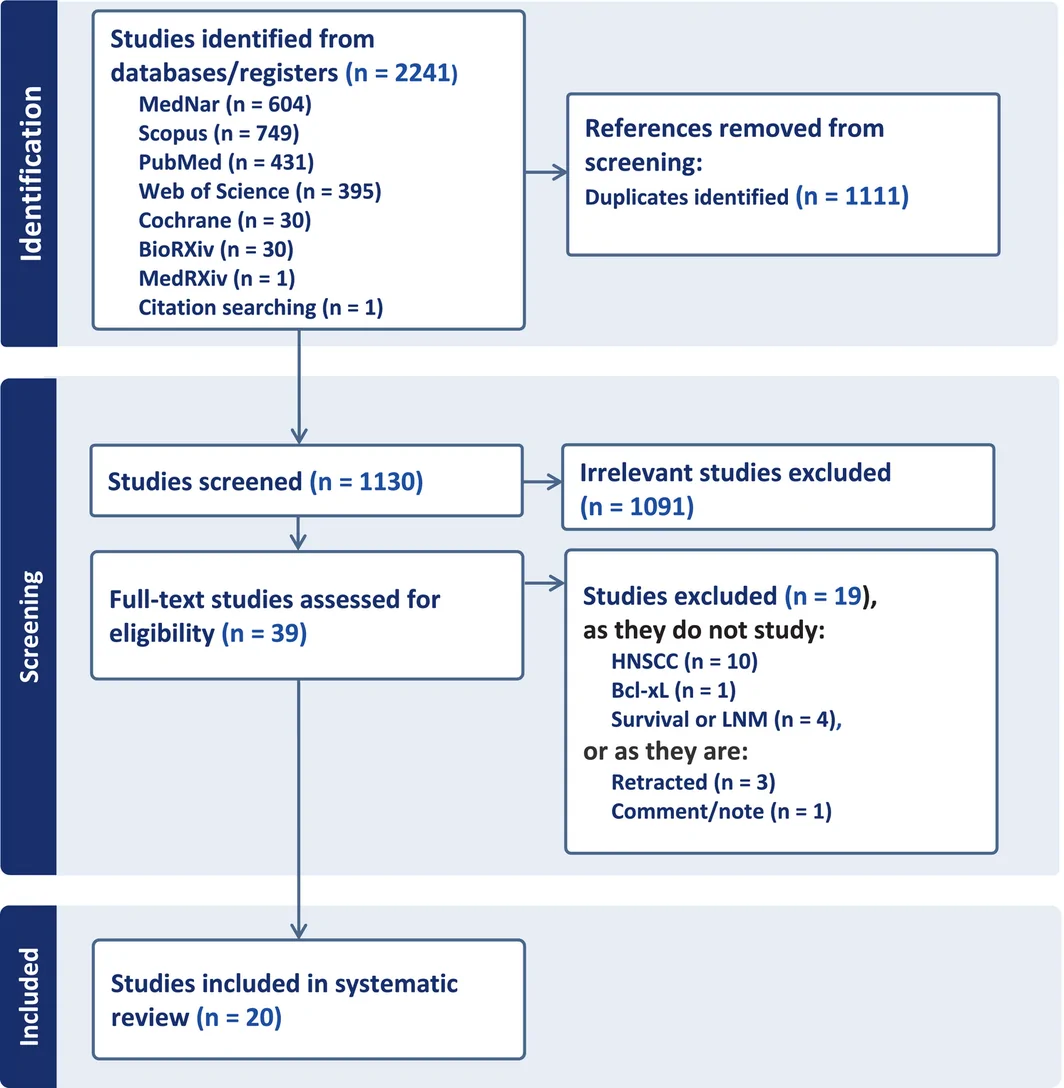
Flowchart of the systematic literature search as depicted in Covidence.

Figure 1 illustrates the workflow of this systematic review. Our search strategy initially retrieved a total of 2241 hits from six databases. After removing duplicates (*n* = 1130) and reports deemed irrelevant based on the titles and/or abstracts (*n* = 1091), we retrieved and analyzed the full text for 39 reports. Finally, 20 studies which provided data on the association between Bcl‐xL expression and patients' prognostic variables were selected for further analysis. An overview of the reports is given in Table 1, including the reference, cancer type, effect on prognosis and presence of metastases, and the statistical analyses used. Additional details such as the antibody used, the Bcl‐xL positivity, and the cut‐off values are presented in Table S1. The studies included were published between 1999 and 2025, with a median publication year falling between 2012 and 2013.

**TABLE 1:** Overview of the reports on Bcl‐xL evaluating HNSCC prognosis and the presence of metastasis.

First author (year)	Tumor location	Total no. of IHC samples	Effect on prognosis	*p*	Effect on metastasis	*p*
			Endpoint (OS/CSS/DFI/DFS/DSS/RFS) and statistical method		LNM/nodal status/nodal disease at presentation/N stage/pN and statistical method	
Carter et al. (2019) [14]	HNSCC	191	OS (Kaplan–Meier)	NS		
Bauer et al. (2007) [17]	HNSCC	55	DSS (NR)	NR		
Pena et al. (1999) [18]	HNSCC	42	OS/EFS/DFS (Kaplan–Meier)	NS/NS/NS	pN (chi‐square)	NS
Thanasan et al. (2023) [19]	LSCC	41	DFS (Kaplan–Meier and Cox regression)	NR		
Chen et al. (2015) [20]	LSCC	84^c^	OS (Kaplan–Meier and Cox regression)	NR	LNM (unpaired *t*‐test and one‐way ANOVA)	NR
Bradford et al. (2014) [21]	LSCC	58	OS/DFS (Cox regression)	NS/NS	N stage (Kruskal–Wallis)	NR
Kumar et al. (2008)^a^ [22]	LSCC	70	OS/DFS (Kaplan–Meier)	NS/NS		
Zhang et al. (2005) [23]	LSCC	45			LNM (Fisher's exact test)	**
Trask et al. (2002) [24]	LSCC	47	OS (Kaplan–Meier)	NS	Nodal status (chi‐square and Fisher's exact test)	NR
Thai et al. (2025) [25]	NPC	174	OS/PFS (Kaplan–Meier and Cox regression)	NS/NS		
Bandoh et al. (2002) [26]	MSSCC	70	OS/DFS (Kaplan–Meier and Cox regression)	NS/NS	Nodal status (Mann–Whitney *U* test, chi‐square, and Fisher's exact test)	NS
Ziai et al. (2019) [15]	OPSCC	166	OS/DSS (Kaplan–Meier and Cox regression)	*(uni)/NS (multi)/NS (uni)	Nodal disease at presentation (chi‐square)	*
Barber et al. (2013) [27]	OPSCC	237	RFS (Kaplan–Meier)	NS	Nodal disease at presentation (chi‐square)	*
Kumar et al. (2008)^b^ [28]	OPSCC	50	OS/DSS (Kaplan–Meier and Cox regression)	NS/NS	Nodal status (Wilcoxon and Kruskal–Wallis)	NR
Alam and Mishra (2021) [29]	OSCC	125			LNM (chi‐square and Fisher's exact test)	NS
Mallick et al. (2013) [30]	OSCC	39	DFS (Kaplan–Meier and Cox regression)	NS	LNM (Mann–Whitney *U* test)	NR
Bose et al. (2012) [31]	OSCC	69	DSS (Kaplan–Meier and Cox regression)	NS	pN (Fisher's exact test)	NS
Camisasca et al. (2009) [32]	OSCC	53	OS/CSS/DFI (Kaplan–Meier and Cox regression)	NR/NR/*	pN (Fisher's exact test)	NS
Baltaziak et al. (2006) [33]	OSCC	56			LNM (chi‐square)	NS
Zhang et al. (2014) [34]	OTSCC	100^d^	OS/DFS/CSS/RFS (Kaplan–Meier and Cox regression)	** */**/*(uni), **(multi)/NS	Nodal status (chi‐square and Fisher's exact test)	*

Abbreviations: ANOVA, analysis of variance; DFS, disease‐free survival; DSS, disease‐specific survival; HNSCC, head and neck squamous cell carcinoma; LNM, lymph node metastasis; LSCC, laryngeal squamous cell carcinoma; MSSCC, maxillary sinus squamous cell carcinoma; NPC, nasopharyngeal carcinoma; NR, not reported; NS, not significant; OPSCC, oropharyngeal squamous cell carcinoma; OS, overall survival; OSCC, oral squamous cell carcinoma; OTSCC, oral tongue squamous cell carcinoma; PFS, progression‐free survival; RFS, recurrence‐free survival.

^a^ Kumar, Cordell, D'Silva, et al. (2008).

^b^ Kumar, Cordell, Lee, et al. (2008).

^c^ Immunoblot.

^d^ 80 squamous cell carcinoma, 20 oral tongue adenocarcinoma.

* *p* < 0.05.

** *p* < 0.01.

The majority of the articles included in our analysis met the quality criteria (mean: 13/16; median: 15/16) assessed using the REMARK [35] (Table S2) and QUIPS [36] (Figure S1) guidelines. Only two articles reported less than half of the recommended REMARK items [17, 23]. All articles reported the studied marker and the study objectives in the introduction (Criteria 1); yet, they most often failed to describe the results for multivariate analyses (Criteria 16). Similarly, for QUIPS, the most presented information covered prognostic and outcome measurements, whereas information on confounding factors and statistical analysis was most often missing. In general, QUIPS showed high bias for the same studies that scored low in REMARK; yet overall, most studies showed low bias (13/20).

### 2.2 Bcl‐xL Protein Was Frequently Expressed in Tumors Without Clear Association With Pathological Parameter

In the majority of the studies (19/20), the Bcl‐xL protein levels were examined using immunohistochemical (IHC) staining, with one exception, which only employed immunoblot analysis [20]. In addition to IHC, one article used immunoblot analysis [14] and three articles also assessed the gene expression with RT‐PCR or Nanostring [25, 29, 34] (Table 1).

The Bcl‐xL protein was commonly detected in HNSCC tumors. Three studies reported positive Bcl‐xL staining in all tumor samples [15, 27, 31] (Table S1). Six articles reported varying Bcl‐xL positivity, ranging from 44.6% to 96.20% of cases (average 59.5%) [18, 23, 25, 26, 32, 33, 34]. A high level of the Bcl‐xL protein was observed in five studies ranging from 47.3% to 74% of the tumor specimens (average 59.1%) [22, 24, 27, 31, 34]. Five studies did not report the precise Bcl‐xL protein levels in the tumor samples [17, 21, 28, 29, 30]. Bcl‐xL staining localized in the cytoplasm of tumor cells [19, 22], in tumor core and in the invasive front of oral, hypopharyngeal, and laryngeal SCCs [14]. Interestingly, Bcl‐xL was more commonly expressed in LNM (63.6%) than in primary OSCC samples (44.6%) [33].

No clear association between any pathological parameters and the Bcl‐xL protein levels in HNSCC tumors emerged. Three studies reported that the Bcl‐xL expression positively associated with the tumor grade (*p* < 0.04 [33], *p* < 0.001 [34], *p* < 0.05 [23]), whereas two studies found no such correlation (*p* > 0.05 [26, 31]). One study reported a positive correlation between the Bcl‐xL expression and TNM stage (*p* < 0.05 [34]), whereas three studies reported no correlation (*p* > 0.05 [23, 24, 31]). OTSCC showed significantly higher Bcl‐xL gene expression and protein levels compared to adenocarcinoma samples of the tongue [34].

### 2.3 Bcl‐xL Levels Have Been Studied Both in Specific HNSCC Subtypes and Mixed Sample Populations

The reports included in this systematic review analyzed samples from different head and neck regions (Table 1). On average, 89 (*n* = 39–237, total 1772) tumor samples were used per study. Six articles included laryngeal squamous cell carcinoma (LSCC; *n* = 41–84) [19, 20, 21, 22, 23, 24], five oral squamous cell carcinoma (OSCC; *n* = 39–125) [29, 30, 31, 32, 33], and three oropharyngeal squamous cell carcinoma (OPSCC) samples (*n* = 50–237) [15, 27, 28]. Three articles studied HNSCC without mentioning a specific region (*n* = 42–191) [14, 17, 18]. Oral tongue carcinoma (of which 80 were OTSCC and 20 adenocarcinoma samples) [34], maxillary sinus squamous cell carcinoma (MSSCC, *n* = 70) [26], and nasopharyngeal carcinoma [25] (NPC, *n* = 174) were examined in singular studies.

### 2.4 Association Between the Bcl‐xL Level and Survival Was Rarely Found

The association between the Bcl‐xL expression levels and patient survival outcomes was explored in 17 studies. Twelve articles studied overall survival (OS), seven disease‐free survival (DFS), three disease‐specific survival (DSS), two recurrence‐free survival (RFS), and one event‐free survival and one progression‐free survival (PFS) (Table 1). Three studies reported a significant association between high Bcl‐xL levels and lower survival outcomes, whereas 11 articles reported no significant association (Table 1). High Bcl‐xL levels in OPSCC patients' samples were associated with a lower OS (*p* = 0.04), but not with DSS (*p* = 0.3) [15]. Similarly, another study [27] reported no association between the Bcl‐xL levels and RFS in OPSCC patients. In the OSCC patients treated with definitive radiotherapy, a significant correlation between a high Bcl‐xL level and a poor DFS in a multivariate analysis (*p* = 0.004) was identified, but statistical significance was not reached in an univariate analysis (*p* = 0.086) [30]. Another report on OSCC patients revealed no association between the Bcl‐xL expression and the 5‐year DSS (*p* = 0.469) [31]. Three studies with LSCC patients [21, 22, 24], a study with MSSCC patients [26], a study with NPC patients [25], and two studies with unspecified HNSCC patients [14, 18] reported no association between the Bcl‐xL levels and OS. Furthermore, a study with NPC patients reported no association between PFS and Bcl‐xL [25]. Interestingly, OTSCC patients with high tumoral Bcl‐xL mRNA and protein expressions experienced significantly shorter OS (*p* = 0.001) and DFS (*p* = 0.002) [34]. In addition, a high Bcl‐xL level was associated with a shorter DSS in an univariate (*p* = 0.017) and multivariate analysis (*p* = 0.009) in that same study [34]. Three studies investigated the association between Bcl‐xL expression and survival, as described in their methods, but did not report *p* values or provide any statement regarding statistical significance [17, 19, 20].

### 2.5 Bcl‐xL Levels Correlate With Lymph Node Metastasis in 40% of Reported Analyses

Fifteen studies claimed to analyze the association between the Bcl‐xL levels and lymph node metastasis (LNM), but only 10 reported the results of these analyses. Four of 10 studies described a significant association (40%, Table 1). Two studies with OPSCC patients reported that a high level of Bcl‐xL associated with a positive LNM at presentation (*p* = 0.039; *p* = 0.04, respectively [15, 27]), but one study did not report their results [28]. Similarly, the Bcl‐xL protein level in LSCC patients' samples significantly associated with LNM (*p* < 0.01) [23]. However, three studies on LSCC did not mention any results for their LNM analysis [20, 21, 24]. One study on MSCC [26], and four on OSCC [29, 31, 32, 33] reported no association with LNM. Finally, tumoral Bcl‐xL level in OTSCC patients with LNM was significantly (*p* < 0.05) higher than that in patients without LNM [34].

### 2.6 Meta‐Analyses Showed That the Tumoral Bcl‐xL Levels Cannot Be Used to Evaluate Overall Survival or Lymph Node Metastasis

**FIGURE 2:**
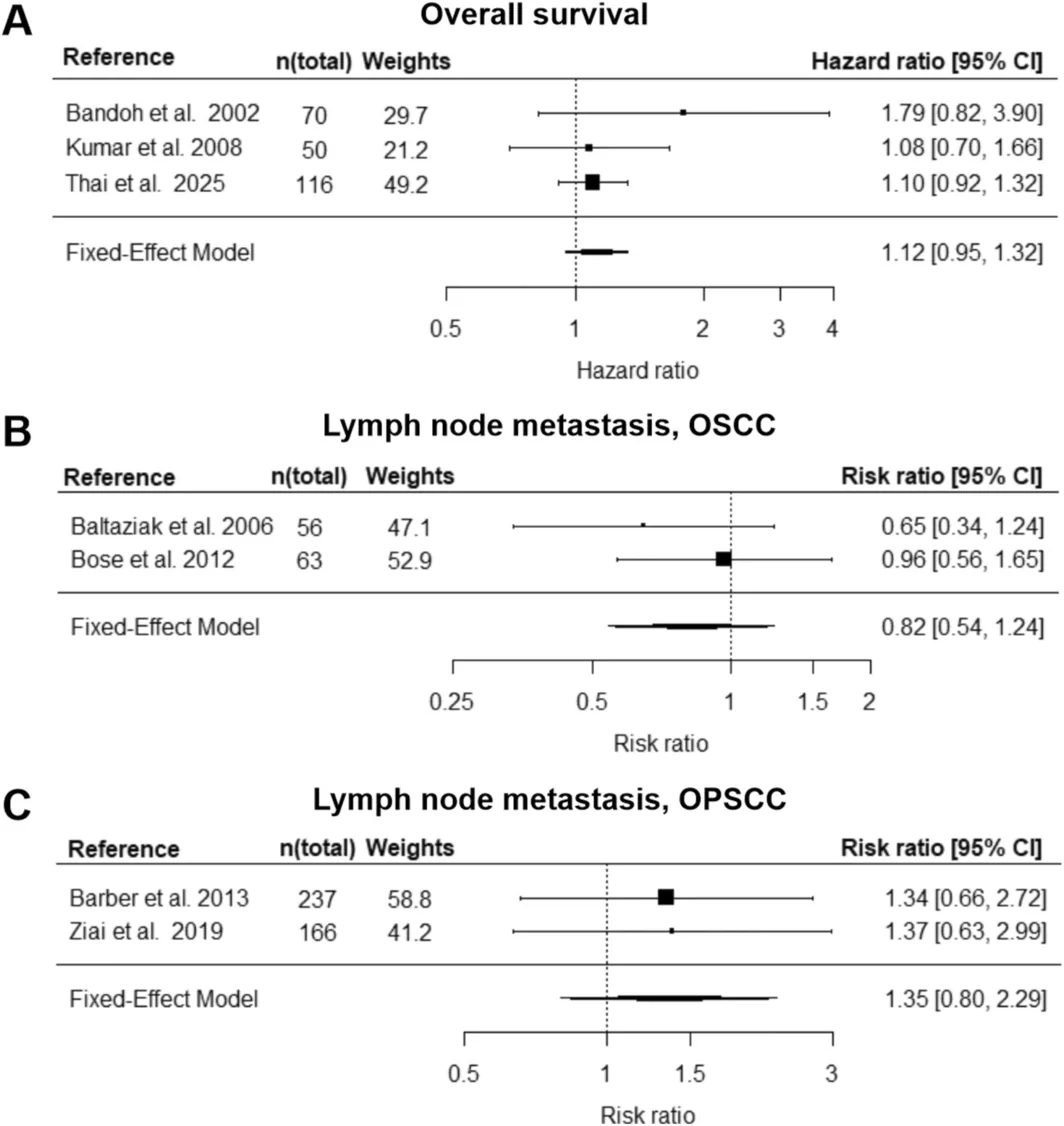
Meta‐analyses of the effect of a high tumoral Bcl‐xL expression level on overall survival (A) or lymph node metastasis (B and C) in different head and neck squamous cell carcinomas presented as forest plots. CI, confidence interval; OPSCC, oropharyngeal squamous cell carcinoma; OSCC, oral squamous cell carcinoma.

We evaluated the usefulness of the Bcl‐xL levels for predicting OS and LNM by combining suitable studies for meta‐analyses. Only three studies for each meta‐analysis were used given the limited information reported in the original publications. A meta‐analysis on OS (MSCC [26], OPSCC [28], NPC [25]) revealed no significant association between a high Bcl‐xL expression level and risk of death (HR 1.12, 95% CI 0.95–1.32, *p* = 0.175, Figure 2A). In addition, a meta‐analysis on LNM in OSCC [31, 33] also did not reach statistical significance (risk ratio 0.82, 95% CI 0.54–1.24; *p* = 0.3453, Figure 2B). Similarly, no significant effect was found for a high Bcl‐xL expression level and LNM when combining studies on OPSCC [15, 27] (risk ratio 1.35, 95% CI 0.80–2.29, *p* = 0.2569, Figure 2C). Low heterogeneity between included studies was found for all meta‐analyses (Cochran's *Q p* values 0.4858, 0.3509, and 0.9601, respectively).

## 3 Discussion

Bcl‐xL is an anti‐apoptotic protein that regulates the intrinsic apoptosis pathway by inhibiting the release of cytochrome c and caspase activation [37]. Bcl‐xL affects the aggressiveness of various tumor cells in vitro, and these effects can be reversed through knockdown or inhibition. For instance, overexpression of Bcl‐xL in melanoma and glioblastoma cell lines led to increased migration, invasion, and tumor sphere formation, which could be reversed by silencing with small interfering RNAs or by applying the Bcl‐xL‐specific inhibitor WEHI‐539 [38]. In our previous studies, we have demonstrated that Bcl‐xL inhibition using A‐1155463, A‐1331852, or navitoclax in vitro led to increased apoptosis and reduced proliferation, clonogenicity, and invasion in HNSCC cells [16, 39]. Moreover, smaller tumors and fewer metastases were observed in a zebrafish larvae model when employing Bcl‐xL inhibition [16]. Furthermore, several clinical trials have been completed on navitoclax for leukemia and some solid tumors [40], but not thus far for any HNSCCs. Clinical trials for HNSCCs using the nonspecific Bcl‐2 inhibitor Gossypol (AT‐101) yielded largely disappointing results: no improvement in PFS or OS was observed in metastatic HNSCC patients treated using a combination of docetaxel and AT‐101 compared with docetaxel alone [41]. Therefore, the drug AT‐101 is no longer manufactured as a drug. Similarly, no improvement in organ preservation was observed in laryngeal SCC patients when AT‐101 was combined with a platinum plus docetaxel treatment regimen [42]. This suggests that novel Bcl‐2 family member‐specific inhibitors may yield more favorable results.

Previous studies reported that the overexpression of Bcl‐xL is associated with a shorter survival in non‐small cell lung cancer, urothelial carcinoma, and follicular lymphoma, respectively [43, 44, 45]. However, we demonstrate that most studies examining the association between tumoral Bcl‐xL protein levels and survival in HNSCC reported no significant correlation, which was also repeated in our OS meta‐analysis. Studies on tumors with unspecified locations (primarily HNSCC and OSCC) revealed nonsignificant results, whereas studies with more specific subsite selection, such as LSCC [23], OPSCC [15], or OTSCC [34], reported significant findings more often. This highlights the importance of investigating HNSCC from different locations separately: the etiologies, tumorigenesis, and characteristics of various subtypes appear highly different.

Studies have suggested that a high Bcl‐xL protein level correlates with LNM in different cancers, such as colorectal and colon cancer as well as thyroid carcinoma [46, 47, 48]. We found a significant association with LNM in four of 10 articles which reported such analyses. These articles studied LSCC, OPSCC (two articles), and OTSCC. Yet, three other studies on LSCC, with close to 200 samples, detected no correlation. Similarly, in an OPSCC study, no correlation was reported. Based on our meta‐analysis, we could not identify a statistically significant association between Bcl‐xL and LNM in OPSCC. This raises questions about the strength of the association between a high Bcl‐xL expression and nodal disease in LSCC and OPSCC. Most OSCC studies either did not report or reported a nonsignificant association between the Bcl‐xL expression levels and LNM, in line with our meta‐analysis. Notably, the association between high Bcl‐xL expression levels and LNM appears most clearly in OTSCC, although this conclusion is greatly limited by the number of studies.

We should also consider whether survival and LNM are the only factors worth assessing. One interesting finding from our systematic review was that several studies reported a clear association between the Bcl‐xL protein level and treatment resistance or organ preservation following treatments. In OSCC patients, high Bcl‐xL correlated with chemoradiation resistance and the severity of the disease [29] as well as poor response to radiotherapy [30]. Furthermore, three reports [17, 22, 24] showed that patients with low tumoral Bcl‐xL levels had higher larynx preservation rates following chemotherapy or irradiation compared with patients with a high Bcl‐xL. Interestingly, patients whose tumors expressed the lowest proportion of Bcl‐xL in relation to induction chemotherapy experienced 100% DFS [17]. Thus, assessing Bcl‐xL levels may prove useful in distinguishing patients who might benefit most from chemoradiation therapy.

Based on our systematic review, tumoral Bcl‐xL expression is unlikely to represent a suitable marker for assessing prognosis (survival or LNM) among HNSCC patients. Yet, focusing on a specific subtype is worth considering, given that HNSCCs vary greatly in terms of characteristics and etiological factors such as concomitant viral infections. Furthermore, viruses may affect Bcl‐xL expression levels as demonstrated by a study in which the latency‐associated transcript (LAT) from HSV‐1 (herpes simplex virus) can reduce apoptosis and induce latency by promoting the accumulation of Bcl‐xL [49]. Interestingly, we identified a promising correlation between both survival and the presence of LNM and a high Bcl‐xL level, although only in one OTSCC study [34]. Furthermore, this study (with a high REMARK score, 16/16) associated high Bcl‐xL level with tumor grade, TNM stage, and tumor progression. These findings indicate that Bcl‐xL overexpression might play a greater role in tumorigenesis of OTSCC versus other HNSCCs and that Bcl‐xL may serve as a useful marker for clinical diagnosis of OTSCC. However, since only one article examined OTSCC patients, it is premature to draw any definitive conclusions, and further multicenter studies with a larger number of OTSCC patients are needed.

To our knowledge, this is the first systematic review and meta‐analysis on the role of Bcl‐xL in HNSCC prognosis. We identified 20 original reports on this topic, which were of sufficiently good quality and low bias based on our assessment using the REMARK and QUIPS criteria. That said, we must point out several limitations of our findings. First, the reports we included focused on different subtypes of HNSCC: the most common subsites studied were LSCC and OSCC, both examined in five studies, but we only identified one report each for MSCC, NPC, and OTSCC patients, respectively. The second limitation lay in the variability in the number of samples (from 39 to 237). Furthermore, studies used several different antibodies for the immunohistochemical analyses and varying cut‐off criteria, complicating comparison across studies (Table S1). In some studies, exact values (*p*‐, hazard, and risk ratio or confidence intervals) were not published, rendering such studies unsuitable for meta‐analyses.

## 4 Materials and Methods

### 4.1 Search Protocol

This systematic review followed the updated Preferred Reporting Items for Systematic Reviews and Meta‐Analyses (PRISMA 2020) guidelines [50]. We registered this study and its protocol in PROSPERO (International Prospective Register of Systematic Reviews) with the registration number CRD42023418710. Searches were performed in Pubmed (https://pubmed.ncbi.nlm.nih.gov/), Scopus (https://www.scopus.com/), Web of Science (https://www.webofscience.com/wos), Cochrane Library Central (https://www.cochranelibrary.com/advanced‐search), MedRXiv (https://www.medrxiv.org/), BioRXiv (https://www.biorxiv.org/), and MedNar (https://mednar.com/mednar/desktop/en/search.html). Our search included all articles published before April 2026. The key concepts used in the search were: Bcl‐xL and cancer (“oral cancer” OR “head and neck cancer”) and prognosis (surviv* OR lymph node metastas*). We modified the search terms due to the limitations in the search query length (Table S3). The terms were searched across all fields (PubMed and Web of Science) or in the titles, abstracts, and keywords (Scopus and Cochrane Library Central). In the preprint servers (MedRXiv, BioRXiv) and databases (MedNar), we relied on the advanced search function.

### 4.2 Review of Relevant Publications

We used the Covidence Systematic Review (Veritas Health Innovation, Melbourne, Australia. Available at www.covidence.org), a web‐based collaboration software platform that streamlines the systematic review process, to screen the studies and create a flowchart of the review process. We originally retrieved a total of 2241 search hits. After removing duplicate articles, 1130 articles remained. Two independent reviewers (L.L. and M.J.) reviewed the title and abstract from each article. Disagreements between the two reviewers were resolved by the third and fourth reviewers (K.J. and K.K.). Reports were excluded if articles were not original research articles (reviews, comments, or letters), or if the study relied only on animal in vivo models or in vitro models. We also excluded studies regarding cancers other than head and neck squamous cell carcinomas (HNSCCs). Following the application of these exclusion criteria, 39 potentially relevant studies remained, and the full text was screened by two reviewers (K.J. and K.K.). Studies were included if the Bcl‐xL gene expression or protein levels were examined using immunohistochemistry (IHC), RNA in situ hybridization, or similar techniques in human tissues, and if the study analyzed the association between the Bcl‐xL expression level and at least one of the following prognostic variables: overall survival (OS), disease‐free survival (DFS), disease‐specific survival (DSS), progression‐free survival (PFS), recurrence‐free survival (RFS), or occurrence of lymph node metastasis in HNSCC. Finally, 20 studies were eligible and included in this systematic review. Results from survival or LNM analyses from the original reports were synthesized in Table 1.

### 4.3 Evaluation of the Publication Quality and Bias

We used the Reporting Recommendations for Tumor Marker Prognostic Studies (REMARK) [35] guideline to assess the publication quality and risk of bias. The quality of each study was evaluated by two independent reviewers (L.L. and M.J.) and synthesized in a table. Disagreements between reviewers were solved by a third person (R.T.). The REMARK guideline consists of 20 criteria, from which we evaluated 16 items. We excluded four items—3, 8, 9, and 18—because these criteria describe the treatments studied (Criteria 3), the candidate variables in the models (Criteria 8), the size of the samples (Criteria 9), and the results from possible further investigations (Criteria 18). These areas were not relevant to the estimates included in this systematic review. The criteria were deemed fulfilled if the study reported most items.

We assessed the potential publication bias using the Quality In Prognosis Studies (QUIPS) tool [36]. The risk of bias was evaluated by two independent reviewers (K.J. and K.K.), who resolved disagreements and synthesized the findings in a table. The criteria were deemed fulfilled if the study reported most items.

### 4.4 Meta‐Analysis on Overall Survival and Risk of Lymph Node Metastasis

The open software R (version 4.4.0) with the package “metafor” (version 4.6–0) was used to perform meta‐analyses. Fixed effects model was chosen to estimate the combined effect of high Bcl‐xL level on overall survival or risk of lymph node metastasis in selected studies. The package “meta” (version 8.0–1) was used to draw forest plots. The variance between included studies was estimated by calculating Cochran's *Q* and *I* ^2^. The *p* < 0.05 is considered statistically significant.

## Author Contributions

L.L. participated in designing the review protocol, conducted the literature searches, screened potentially eligible studies, extracted the data, compiled the summary on the extracted data, and assisted in drafting the first version of the manuscript. L.L., M.J., K.J., and K.K. performed the study quality or bias analyses. R.T. acted as a referee for the quality analyses, performed meta‐analyses, and participated in finalizing the manuscript. M.J. participated in designing the review protocol, conducted the literature searches, screened potentially eligible studies, extracted the data, compiled the summary on the extracted data, and reviewed the manuscript. T.S. and P.Å. secured funding for this study, participated in the project design and supervision as well as finalizing the manuscript. K.J. participated in designing the review protocol and the methods we employed, provided methodological and scientific guidance, interpreted the results, and drafted the first version of the manuscript. K.K. participated in designing the project and the review protocol, published the review protocol (PROSPERO), provided methodological and scientific guidance, interpreted the results, and drafted the first version of the manuscript.

## Funding

This work was supported by the Suomen Hammaslääkäriseura Apollonia (Grant 20240038), the Sigrid Juséliuksen Säätiö (Grant 8136), the Helsingin ja Uudenmaan Sairaanhoitopiiri (Grants TYH2024308 and TYH2023333), the Syöpäsäätiö (Grant 22.11.2023), the Oulun Yliopistollinen Sairaala (Grant K43712), the Minerva Foundation (Grant 181), and the Academy of Finland (Grant 354669).

## Conflicts of Interest

The authors declare no conflicts of interest.

## Supporting information


**Figure S1:** Quality assessment of the included studies using the QUIPS tool. The color of each circle indicates the risk of bias: green = low risk, yellow = moderate risk, and red = high risk.

**Table S1:** Detailed description of reports included in the systematic review.
**Table S2:** Quality of reports included evaluated using the REMARK criteria.

**Table S3:** Databases and search terms used in this study.

## Data Availability

The data that support the findings of this study are available from the corresponding author upon reasonable request.
