# Supplementary material for: Prognostic Value of Bcl‐xL in Head and Neck Squamous Cell Carcinoma: A Systematic Review and Meta‐Analysis
Source: J Oral Pathol Med. 2026 Jun 13;55(8):885–94. doi: 10.1111/jop.70155 (PMC13533851; doi:10.1111/jop.70155)
Supplement: Supplementary file 1 [file JOP-55-885-s001.docx]

**Supplementary Table 1.** Detailed description of reports (included in the systematic review)

| First author (Year) | **Tumor location** | **Total no of IHC samples** | **Antibody, manufacturer** | **Bcl-xL expression in tumor / LNM** | **Expression cutoff** |
| --- | --- | --- | --- | --- | --- |
| Alam & Mishra 2021 | OSCC | 125 | Monoclonal antibody  (sc-8392, Santa Cruz) | NA | NA |
| Baltaziak et al. 2006 | OSCC | 56 | Polyclonal antibody for Bcl-xL (Santa Cruz) | 44.6% / 63.6% | >10% cells stained positive |
| Bandoh et al. 2002 | MSSCC | 70 | Anti-Bcl-xL polyclonal antibody | 47% | >30% of cells stained positive |
| Barber et al. 2013 | OPSCC | 237 | Monoclonal anti-Bcl-xL  (Cell Signaling Technology) | All samples positive; high expression of Bcl-xL was demonstrated in 47.3% of tumor specimens. | Bcl-xL values that were above the median value calculated for the entire cohort of 226 specimens were deemed positive. |
| Bauer et al. 2007 | HNSCC | 55 | Ab-2, clone 7D9 (Lab Vision) | NA | NA |
| Bose et al. 2012 | OSCC | 69 | Rabbit monoclonal anti-Bcl-xL (Cell Signaling Technology) | Bcl-xL was strongly expressed in all OSCC samples and highly expressed in 49% of samples compared to control tissue. | Median Bcl-xL expression in the OSCC tumor samples was 3450 [95% CI 3248–3705], which approached the upper limit of Bcl-xL expression in the normal OCSE 95% CI. We defined this as borderline overexpression. |
| Bradford et al. 2014 | LSCC | 58 | Bcl-xL (Ab-2, clone 7D9, MS-1334-PO, Lab Vision) | NA | Tumor cells expressing 1: <5% staining; 2: 5–20% staining; 3: 21–50% staining; and 4: 51–100% staining. Intensity 1–4 (none to strong). |
| Camisasca et al. 2009 | OSCC | 53 | Monoclonal anti-Bcl-xL, clone A35-10 (DAKO) | 96.2% | 0.141 (% of total area) |
| Carter et al. 2019 | HNSCC | 191 | IHC: Bcl-xL (Cell Signaling Technology 2764)  WB: Bcl-xL (sc-8392, Santa Cruz) | Bcl-xL expression was significantly elevated in the tumor core and advancing in front of oral, hypopharyngeal, and laryngeal HNSCCs compared with adjacent normal tissues. | NA |
| Chen et al. 2015 | LSCC | 84^†^ | Bcl-xL (Cell Signaling Technology) | Bcl-xL was upregulated in tumor samples (p < 0.05). | Not relevant |
| Kumar et al. 2008^a^ | LSCC | 70 | Ab2, clone 7D9 (Lab Vision) | High Bcl-xL expression in 71.4%. | <21% (low expression), ≥21% (high expression) |
| Kumar et al. 2008^b^ | OPSCC | 50 | Bcl-xL / 7D9 (Lab Vision) | NA | For Bcl-xL, a scale of 1 to 4 was used: 1 <5%; 2, 5–20%; 3, 21–50%; and 4, 51–100% tumor staining. Intensity was scored as 1 representing no staining; 2, low intensity; 3, moderate; and 4, high intensity. |
| Mallick et al. 2013 | OSCC | 39 | Anti-Bcl-xL (Cell Signaling Technology) | NA | The values above median, high expression; less than median, low expression. Bcl-xL: 60% of cells. |
| Pena et al. 1999 | HNSCC | 42 | Likely anti-Bcl-xL mAb (7B2), i.e., non-commercial antibody | 52% | 4-tiered scale (negative, equivocal, positive, and strongly positive). |
| Thai et al. 2025 | NPC | 174 | Bcl-xL (54H6) rabbit monoclonal antibody (Cell Signaling Technology) | Most NPC samples stained positively for BCL-xL. | No predefined cutoff; BCL-xL quantified using H-score (0–300) and treated as a continuous variable. |
| Thanasan et al. 2023 | LSCC | 41 | Bcl-xL (54H6) rabbit monoclonal antibody (Cell Signaling Technology) | NA | Cutoff determined by ROC curve based on H-score; specific Bcl-xL threshold not reported. |
| Trask et al. 2002 | LSCC | 47 | Bcl-xL (F126, Santa Cruz) | High Bcl-XL expression in 74% of tumor samples. | <2% (no expression); 2–50% (low expression); >51% (high expression). |
| Zhang et al. 2014 | OTSCC | 100^#^ | Anti-Bcl-xL (ab15274, Abcam) | 64% positive samples, high expression in 54%. | The percentage of positive tumor cells was graded as follows: 0, none; 1, 1–25%; 2, 26– 50%; 3, 51–75%; and 4, 76–100%. The intensity of the tumor cells was rated as follows: 0, none; 1, weak; 2, moderate; and 3, intense. |
| Zhang et al. 2005 | LSCC | 45 | Monoclonal mice anti-human Bcl-xL antibody (non-commercial) | 53% | At least 1 after multiplication (i.e., at least faint staining in 1/3 [33%] of cells):  intensity in 4-tiered scale (little to strong) multiplied by the amount (1, <1/3 of cells; 2, 1/3–2/3 of cells; 3, ≥2/3). |
| Ziai et al. 2019 | OPSCC | 166 | Rabbit monoclonal anti-Bcl-xL (Cell Signaling Technology) | The entire cohort was deemed positive for Bcl-xL. | Above median (median undefined). |

^a^Kumar, Cordell, D’Silva, et al., 2008

^b^Kumar, Cordell, Lee, et al., 2008

**Abbreviations:** CI, confidence interval; HNSCC, head and neck squamous cell carcinoma; LSCC, laryngeal squamous cell carcinoma; MSSCC, maxillary sinus squamous cell carcinoma; NA, not available; NPC, nasopharyngeal carcinoma; OPSCC, oropharyngeal squamous cell carcinoma; OSCC, oral squamous cell carcinoma; OTSCC, oral tongue squamous cell carcinoma.

^†^Immunoblot

^#^80 squamous cell carcinoma, 20 oral tongue adenocarcinoma.

**Supplementary Table 2**. Quality of reports included evaluated using the REMARK criteria.

|  | | Criteria | | | | | | | | | | | | | | | | |
| --- | --- | --- | --- | --- | --- | --- | --- | --- | --- | --- | --- | --- | --- | --- | --- | --- | --- | --- |
| Reference | **Cancer** | **1** | **2** | **4** | **5** | **6** | **7** | **10** | **11** | **12** | **13** | **14** | **15** | **16** | **17** | **19** | **20** | **Score**  **(x/16)** |
| Alam & Mishra 2021 | OSCC | x | x | x | x |  | x | x | x | x | x | x | x |  | x | x | x | **14/16** |
| Baltaziak et al. 2006 | OSCC | x |  | x | x |  | x |  | x | x |  |  |  |  | x | x |  | **8/16** |
| Bandoh et al. 2002 | MSSCC | x | x | x | x | x | x | x | x | x | x | x | x | x | x | x | x | **16/16** |
| Barber et al. 2013 | OPSCC | x | x | x | x | x | x | x | x | x | x | x | x | x | x | x | x | **16/16** |
| Bauer et al. 2007 | HNSCC | x |  | x |  |  |  |  |  |  |  |  |  |  |  | x |  | **3/16** |
| Bose et al. 2012 | OSCC | x | x | x | x | x | x | x | x | x | x | x | x | x | x | x | x | **16/16** |
| Bradford et al. 2014 | LSCC | x | x | x | x | x | x | x | x | x |  | x | x | x | x | x | x | **15/16** |
| Camisasca et al. 2009 | OSCC | x | x | x | x | x | x | x | x | x | x | x | x | x | x | x | x | **16/16** |
| Carter et al. 2019 | HNSCC | x | x | x | x |  | x | x | x |  | x | x | x |  | x | x | x | **13/16** |
| Chen et al. 2015 | LSCC | x | x | x | x | x | x | x | x | x | x | x | x | x | x | x | x | **16/16** |
| Kumar et al. 2008^a^ | LSCC | x | x | x | x |  | x | x | x | x |  | x | x |  | x | x | x | **13/16** |
| Kumar et al. 2008^b^ | OPSCC | x | x |  | x | x | x | x | x | x | x | x | x | x | x | x | x | **15/16** |
| Mallick et al. 2013 | OSCC | x | x |  | x |  | x | x | x | x | x | x | x | x | x | x | x | **14/16** |
| Pena et al. 1999 | HNSCC | x | x | x | x | x | x | x | x | x | x | x | x |  | x | x | x | **15/16** |
| Thai et al. 2025 | NPC | x | x | x | x | x | x | x | x | x | x | x | x | x | x |  | x | **15/16** |
| Thanasan et al. 2023 | LSCC | x | x | x | x | x | x | x | x | x | x | x | x |  | x | x | x | **15/16** |
| Trask et al. 2002 | LSCC | x | x | x | x | x | x |  | x | x | x | x | x |  | x | x | x | **14/16** |
| Zhang et al. 2014 | OTSCC | x | x | x | x | x | x | x | x | x | x | x | x | x | x | x | x | **16/16** |
| Zhang et al. 2005 | LSCC | x |  |  |  |  |  |  |  |  |  |  |  |  |  |  |  | **1/16** |
| Ziai et al. 2019 | OPSCC | x | x | x | x | x | x |  | x | x | x | x | x | x | x | x | x | **15/16** |
|  |  |  |  |  |  |  |  |  |  |  |  |  |  |  |  |  |  |  |
|  | **No of articles per Criteria** | **20** | 17 | 17 | 18 | 13 | 18 | 15 | 18 | 17 | 15 | 17 | 17 | **11** | 18 | 18 | 17 |  |

**Abbreviations:** HNSCC, head and neck squamous cell carcinoma; LSCC, laryngeal squamous cell carcinoma; MSSCC, maxillary sinus squamous cell carcinoma; NA, not available; NPC, nasopharyngeal carcinoma; OPSCC, oropharyngeal squamous cell carcinoma; OSCC, oral squamous cell carcinoma; OTSCC, oral tongue squamous cell carcinoma.

^a^Kumar, Cordell, D’Silva, et al., 2008

^b^Kumar, Cordell, Lee, et al., 2008
