# Supplementary material for: Prognostic Value of Bcl‐xL in Head and Neck Squamous Cell Carcinoma: A Systematic Review and Meta‐Analysis
Source: J Oral Pathol Med. 2026 Jun 13;55(8):885–94. doi: 10.1111/jop.70155 (PMC13533851; doi:10.1111/jop.70155)
Supplement: Supplementary file 2 [file JOP-55-885-s002.docx]

**Supplementary Figure 1.** Quality assessment of the included studies using the Quality in Prognosis Studies (QUIPS) tool. The color of each circle indicates the risk of bias: green = low risk, yellow = moderate risk, and red = high risk.
