# Supplementary material for: Prognostic Value of Bcl‐xL in Head and Neck Squamous Cell Carcinoma: A Systematic Review and Meta‐Analysis
Source: J Oral Pathol Med. 2026 Jun 13;55(8):885–94. doi: 10.1111/jop.70155 (PMC13533851; doi:10.1111/jop.70155)
Supplement: Supplementary file 3 [file JOP-55-885-s003.docx]

**Supplementary Table 3**. Databases and search terms used in this study.

**Scopus**

(TITLE-ABS-KEY ( ( head OR neck OR oral OR esophageal OR mouth OR gingival OR lip OR lips OR palatal OR "Salivary Gland*" OR tongue OR otorhinolaryngologic OR laryngeal OR nose OR pharyngeal OR tracheal OR hypopharyngeal OR nasopharyngeal OR oropharyngeal OR tonsillar ) AND ( neoplasm* OR cancer OR carcinoma* ) ) AND TITLE-ABS-KEY ( bcl-xl* OR bcl2l1 OR "bcl-2-like protein 1" OR bclx ) AND TITLE-ABS-KEY ( surviv* OR "lymph node metastas*" OR lnm OR "lymphatic metastas*") )

**PubMed**

**((((((((((("Head and Neck Neoplasms"[Mesh:NoExp]) OR "Esophageal Neoplasms"[Mesh]) OR "Gingival Neoplasms"[Mesh]) OR "Lip Neoplasms"[Mesh]) OR "Palatal Neoplasms"[Mesh]) OR "Tongue Neoplasms"[Mesh]) OR "Otorhinolaryngologic Neoplasms"[Mesh]) OR "Squamous Cell Carcinoma of Head and Neck"[Mesh]) OR "Tracheal Neoplasms"[Mesh]) OR (( head[Text Word] OR neck[Text Word] OR oral[Text Word] OR esophageal[Text Word] OR mouth[Text Word] OR gingival[Text Word] OR lip[Text Word] OR lips[Text Word] OR palatal[Text Word] OR "Salivary Gland*"[Text Word] OR tongue[Text Word] OR otorhinolaryngologic[Text Word] OR laryngeal[Text Word] OR nose[Text Word] OR pharyngeal[Text Word] OR tracheal[Text Word] OR hypopharyngeal[Text Word] OR nasopharyngeal[Text Word] OR oropharyngeal[Text Word] OR tonsillar [Text Word]) AND ( neoplasm*[Text Word] OR cancer[Text Word] OR carcinoma* [Text Word]))) AND (("bcl-X Protein"[Mesh]) OR (bcl-xl*[Text Word] OR bcl2l1[Text Word] OR "bcl-2-like protein 1"[Text Word] OR bclx[Text Word]))) AND ((("Survival"[Mesh]) OR "Lymphatic Metastasis"[Mesh]) OR (surviv*[Text Word] OR "lymph node metastas*"[Text Word] OR lnm[Text Word] OR "lymphatic metastas*"[Text Word]))**

**Cochrane / Web of Science**

(head OR neck OR oral OR esophageal OR mouth OR gingival OR lip OR lips OR palatal OR "Salivary Gland*" OR tongue OR otorhinolaryngologic OR laryngeal OR nose OR pharyngeal OR tracheal OR hypopharyngeal OR nasopharyngeal OR oropharyngeal OR tonsillar) AND ( neoplasm* OR cancer OR carcinoma* ) AND ( bcl-xl* OR bcl2l1 OR "bcl-2-like protein 1" OR bclx ) AND ( surviv* OR "lymph node metastas*" OR lnm OR "lymphatic metastas*")

**BioXRiv / MedRxiv**

bcl-xl AND ("oral cancer" or "head and neck cancer") AND (surviv* OR lymph node metastas*)

**MedNar**

bcl-xl AND ("oral cancer" or "oral squamous cell carsinoma" or "head and neck cancer" or "head and neck squamous cell carsinoma") AND (surviv* OR lymph node metastas*)
